# 肿瘤侵袭转移相关microRNAs的研究进展

**DOI:** 10.3779/j.issn.1009-3419.2010.02.13

**Published:** 2010-02-20

**Authors:** 晓峰 陈, 和勇 王

**Affiliations:** 200433 上海, 同济大学附属上海市肺科医院胸外科 Department of Thoracic Surgery, Shanghai Pulmonary Hospital Affiliated to Tongji University, Shanghai 200433, China

近年来大量研究表明microRNAs(miRNAs)与人类多种肿瘤的发生发展及侵袭转移存在着密切关系, miRNAs可能成为一类新的致癌基因或抑癌基因, 它们通过抑制靶mRNA翻译或诱导靶mRNA降解在转录后水平调控基因表达, 具有癌基因或抑癌基因的功能, 参与肿瘤的发生、发展及侵袭转移。

肿瘤的侵袭转移是指癌细胞从原发部位转移到远处部位并在远处部位形成肿瘤的过程^[1, 2]^。肿瘤侵袭转移是一复杂的、多因素的、多步骤的生物学过程。绝大多数肿瘤患者死亡是由肿瘤侵袭转移引起的, 肿瘤侵袭转移不仅是病情恶化的标志, 而且是治疗失败与死亡的重要原因。目前对肿瘤侵袭转移进行了大量研究与探索, 但其确切机制尚未明确; 随着人们对miRNAs研究不断深入, 已证明miRNAs在肿瘤侵袭转移调控中发挥着重要作用。

## 1 miRNAs生物学特性

miRNAs是一类新近发现的长度约22 nt的非编码小分子RNA, 广泛存在于真核生物体内。Lee等^[3]^于1993年首次在秀丽新小杆线虫突变体内发现第一个miRNA, 命名为lin-4, 其调节线虫细胞发育时序。此后, 在果蝇、拟南芥、斑马鱼、水稻甚至人类细胞中发现大量的类似小分子RNA, 即miRNAs。其现已成为研究RNA的一大热点。miRNAs在转录后水平调控靶基因表达, 具有广泛的基因调节功能^[4]^, 可调节基因活动各个层面, 如生长、分化、凋亡等。据估计人类中存在1 000余种miRNAs, 约1/3基因表达受miRNAs调控^[5]^。

miRNAs具有保守性、表达时空性等特性。成熟的miRNAs通过以下方式发挥调控作用:miRNAs大多与其靶mRNA的3′非翻译区(untranslated region, UTR)一定程度的互补配对, 如果互补配对程度高(大多数植物中), 则可导致靶基因mRNA降解; 如果互补配对程度低(大多数动物中), miRNAs抑制靶基因mRNA翻译^[6-9]^。研究^[10]^还表明miRNAs可调节其靶基因mRNA快速脱腺苷化, 导致mRNA快速衰减和表达水平降低, 进而发挥调控作用。此外, miRNAs还可结合于靶基因开放阅读框或5'非翻译区(5'UTR)。Duursma等^[11]^研究发现miR-148与DNMT3b mRNA编码区结合, 抑制表达; 还有研究^[12]^发现MicroRNA-10a可与核糖体蛋白mRNA的5'非翻译区结合。

研究证明miRNAs参与生命过程中一系列重要进程, 包括早期胚胎发育、细胞增殖、细胞凋亡、细胞死亡、脂肪代谢、甚至肿瘤发生、发展和侵袭转移等。其中miRNAs在肿瘤侵袭转移方面研究越来越多, 一些miRNAs作为原癌基因或抑癌基因已被证实, 这对理解miRNAs在肿瘤侵袭转移中的作用及寻找肿瘤特异性诊断标志物和肿瘤治疗新靶点提供了依据。

## 2 肿瘤侵袭转移中miRNAs的调节功能

肿瘤侵袭转移是一个多因素、多步骤的复杂过程, 涉及癌基因、抑癌基因、信号转导基因、粘附相关分子、蛋白水解酶及众多细胞因子和调节因子等。研究发现miRNAs不仅调控肿瘤细胞增殖, 而且参与肿瘤侵袭转移。miRNAs在肿瘤侵袭转移中可发挥促进或抑制作用。

### 2.1 促进肿瘤侵袭转移的miRNAs

#### 2.1.1 miR-10b

Ma等^[13]^证明miRNA-10b与乳腺癌细胞侵袭性密切相关。miRNA-10b的上调可增加肿瘤细胞运动性和侵袭力, 促进肿瘤侵袭转移, 但不影响细胞活力和增殖。为了探索miRNA-10b在体内能否促进转移, 他们将miRNA-10b引入两种非转移性人类乳腺癌细胞系中(SUM149和SUM159), 然后将这些过表达miRNA-10b的乳腺癌细胞系注射到小鼠乳房脂肪细胞中。结果在植入过表达miRNA-10b的SUM149细胞系的小鼠肺部发现微小转移灶, 在植入过表达miRNA-10b的SUM159细胞系的小鼠中, 80%出现明显肺部转移, 30%还同时有明显腹膜转移, 而对照组没有发现转移灶。Ma等^[13]^还阐明了miRNA-10b调节肿瘤侵袭转移的机制, miRNA-10b在转录因子Twist的诱导下过表达, 进而抑制其靶基因*HOXD10*(一种抑瘤蛋白)的表达, 从而增加转移相关蛋白RHOC的翻译。已有研究^[14, 15]^证明, HOXD10过表达可抑制RHOC蛋白的表达, 进而可以阻止miRNA-10b诱导的肿瘤细胞发生侵袭转移。以上这些研究说明miRNA-10b过表达可以促进乳腺癌细胞发生侵袭转移, 然而miRNA-10b过表达在其它恶性肿瘤尤其是在恶性胶质瘤和胰腺癌等较高侵袭性肿瘤类型中, 能否具有类似作用还有待探索^[16, 17]^。

#### 2.1.2 miR-373和miR-520c

已有明确证据揭示miRNA-373和miRNA-520c也可以促进肿瘤转移。miRNA-373和miRNA-520c是具有相似序列的miRNAs家族, 可促进细胞侵袭和转移。miRNA-373最初作为一种癌基因被认可, 通过抑制p53起到致癌作用^[18]^。Huang等^[19]^将设计好的高表达miRNA-373或miRNA-520c的非转移性人类乳腺癌细胞MCF-7通过尾静脉注入免疫耗竭的小鼠体内后发现在小鼠骨骼、脑和肺都有转移灶, 而对照组没有发现转移。miRNA-373和miRNA-520c主要是通过抑制其靶分子透明质烷(HA)表面受体CD44蛋白(一种转移抑制蛋白)的表达而促进肿瘤细胞侵袭和转移。实验也证明CD44表达下调时非转移性MCF-7细胞变为转移性细胞, miRNA-373的表达在转移灶组织中比原位癌中明显升高, 预示miRNA-373可能成为乳腺癌转移早期诊断指标。最近在前列腺癌的研究^[20]^中, 也进一步证实miRNA-373和miRNA-520c通过调节CD44发挥促进转移的作用。

#### 2.1.3 miR-21

miRNA-21已被证实在多种肿瘤中高表达, 其不仅在肿瘤发生过程中类似致癌基因, 而且还可促进肿瘤侵袭转移。miRNA-21主要通过抑制*PTEN*、*PDCD4*、*TPM1*、*Maspin*和*SPRY2*等靶基因来促进肿瘤发生和侵袭转移的, 所有这些靶基因都是负向调控肿瘤生长和侵袭转移的^[21-26]^。Meng等^[27]^实验结果显示miRNA-21在肝癌细胞中比正常细胞高9倍, 还揭示miRNA-21通过下调PTEN的表达而上调PI3K通路进而促进肿瘤细胞增殖转移。Roldo等^[28]^运用基因芯片技术证实miR-21与胰腺癌的肝转移呈正相关, 这与miRNA-21作用于PDCD4有关。在转移性乳腺癌细胞系MDA-MB-231中, 通过一定方法抑制miRNA-21或增加TPM1的表达都能明显减弱乳腺癌细胞的侵袭转移。在乳腺癌病理标本中Maspin蛋白含量与miRNA-21负相关。Sayed等^[26]^也通过实验证明miRNA-21通过下调靶基因*SPRY2*的表达促进细胞侵袭转移。

#### 2.1.4 miR-155

miRNA-155在侵袭性乳腺癌组织中高表达。Kong等^[29]^证实miRNA-155的表达缺失可抑制TGF-β诱导的上皮间质转化(epithelial mesenchymal transition, EMT), 降低侵袭转移力; 进一步研究发现miRNA-155通过下调其靶基因RhoA的表达, 而促进肿瘤侵袭转移。Gironella等^[30]^也证实胰腺导管癌过表达miR-l55可抑制另一靶基因*TP53INP1*表达, 促进肿瘤侵袭转移。

#### 2.1.5 miR-182

最近研究发现, miRNA-182可以促进黑色素瘤侵袭转移。Segura等^[31]^通过实验证实miRNA-182高表达在体内外均可促进侵袭转移, 在黑色素细胞中下调miRNA-182可阻止细胞侵袭转移并引发细胞凋亡。他们还证实miRNA-182通过直接抑制FOXO3和小眼球相关转录因子而促进侵袭转移, 增加FOXO3和小眼球相关转录因子的表达, 还可抑制miRNA-182的促侵袭转移效果。

### 2.2 抑制肿瘤侵袭转移的miRNAs

#### 2.2.1 miR-335、miR-126和miR-206

在人类乳腺癌中, miRNA-335、miRNA-126和miRNA-206被认为是肿瘤转移抑制因子。Tavazoie等^[32]^发现这三种miRNAs的正常表达能很大程度地降低乳腺癌细胞的转移能力。他们发现在高转移性乳腺癌癌细胞MDA-MB-231中上述三种miRNAs缺失或低表达; 而经诱导高表达这三种miRNAs的人类乳腺癌细胞转移和增殖能力明显降低。其抑制肿瘤转移的机制为:miRNA-126主要通过抑制肿瘤细胞的生长和增殖; miRNA-335和miRNA-206主要影响细胞转移到肺和骨的能力, 也证实miRNA-335可调控转录因子SOX4和细胞外基质中钙粘蛋白C(TNC)而抑制肿瘤转移。

#### 2.2.2 Let-7

Let-7是较早发现的能够抑制肺癌侵袭转移的miRNAs家族。已证实Let-7在高转移性非小细胞肺癌细胞系(non-small cell lung cancer, NSCLC)中表达下降^[33, 34]^。Let-7 miRNAs家族过表达可抑制肿瘤细胞分化、增殖及侵袭转移能力。Let-7主要通过抑制其靶基因*HMGA2*(高迁移率组A蛋白)、RAS家族等抑制肿瘤细胞侵袭转移。

#### 2.2.3 miR-200

许多研究^[35-39]^证实EMT是肿瘤侵袭转移的一个重要步骤, 而很多miRNAs参与EMT调节。miRNA-200家族(miRNA-200a、miRNA-200b、miRNA-200c、miRNA-141和miRNA-429)被认为是一种上皮细胞标记物和EMT的主要调节剂^[40]^。miRNA-200家族在缺失上皮标志物E-catherin蛋白而高表达间叶标记物波形蛋白的侵袭性乳腺癌细胞系中缺失^[41]^, 说明miRNA-200家族可通过调节EMT来抑制肿瘤侵袭转移。其还可以通过调节肿瘤侵袭转移相关因子(TGFβ1、ZEB1/2)来调节肿瘤侵袭转移。近期研究^[42, 43]^发现不仅miRNA-200可下调ZEB1/2的表达, 而且ZEB1/2也可反过来下调miRNA-200家族, 这样在miRNA-200家族和ZEB1/2之间就形成一个互反馈回路。

#### 2.2.4 miR-146a

细胞信号转导通路Rho/Rock是与肿瘤转移相关的重要通路, 其主要通过细胞外基质中的透明质烷(HA)与其受体CD44结合产生应答反应促进肿瘤侵袭转移。Lin等^[44]^研究发现miRNA-146a通过抑制该通路不仅发挥抑癌基因作用, 还介导其靶基因Rock1沉默来抑制肿瘤侵袭转移。

#### 2.2.5 miR-34a

miRNA-34a在多方面推测不仅具有抑癌基因的作用, 而且参与肿瘤细胞的扩散、迁徙和转移。Li等^[45]^研究表明在肝癌细胞中miRNA-34a可下调靶基因c-MET的mRNA水平和蛋白水平, 从而减少c-MET诱导的细胞外信号调节激酶(ERK1/2)。可见miRNA-34a通过调节c-MET信号通路抑制肿瘤细胞侵袭转移。

#### 2.2.6 miR-101

EZH2在侵袭性的实体瘤中高表达(机制尚未明确), 已有研究^[46]^证明在人类前列腺癌细胞中miRNA-101缺失可导致靶基因*EZH2*过表达, miRNA-101负调控靶基因*EZH2*而抑制肿瘤侵袭转移。

#### 2.2.7 miR-31

Valastyan等^[47]^研究发现miRNA-31的表达与人类乳腺癌侵袭转移负相关, miRNA-31过表达可以抑制乳腺癌细胞侵袭转移, 他们采用一种稳定的miRNAs检测方法证实miRNA-31在体内外均可抑制转移, 并且通过阻止miRNA-31在体内外的表达, 使得非侵袭乳腺癌细胞发生转移, miRNA-31通过抑制一系列促转移基因抑制肿瘤侵袭转移, 包括*Fzd3*、*ITGA5*、*M-RIP*、*MMP16*、*RDX*和*RhoA*。后来他们又通过实验在mRNA水平证实ITGA5、RDX、RhoA在体内外均可以影响侵袭能力^[48]^。

#### 2.2.8 miR-138

Liu等^[49]^研究发现miRNA-138可抑制头颈部鳞状上皮细胞癌侵袭转移和促进凋亡, 他们使用miRNAs微点阵法发现一些具有差异表达的miRNAs, 其中miRNA-138是在高转移细胞中减少的一种miRNA, 通过实时定量RT-PCR进行了验证。他们在细胞中转染miRNA-138而抑制了肿瘤细胞侵袭能力, 导致细胞周期受阻和促进凋亡, 还证实敲除miRNA-138则增强肿瘤细胞侵袭能力和抑制凋亡。

#### 2.2.9 miR-145

Sachdeva等^[50]^研究发现miRNA-145不仅能够抑制肿瘤生长, 而且可抑制肿瘤细胞侵袭转移, 他们证实miRNA-145可抑制侵袭性乳腺癌细胞系MDA-MB-231和LM2-4142的侵袭转移能力, 在MDA-MB-231细胞中miRNA-145导致其侵袭能力下调50%, 在LM2-4142中, 其侵袭能力可降低75%, 而把anti-miR-145转染到两个细胞株后, 其侵袭能力均上调。他们还通过实验动物模型证实miRNA-145在体内同样可以抑制肿瘤转移。通过Western blot和免疫荧光等方法证实MUC1作为miRNA-145的一个靶基因可促进细胞侵袭转移, 其可被miRNA-145下调。

## 3 结语

迄今为止, 还有许多与肿瘤相关的miRNAs未被发现, 许多miRNAs的功能及其在肿瘤侵袭转移中的机制还有待揭示。每个miRNA的靶基因不止一个, 其在肿瘤侵袭转移中相互作用还有待研究。但是miRNAs作为一种内源性小分子RNA, 依靠其独特的转录后调节机制不仅调节机体正常生理过程, 而且广泛参与肿瘤发生、发展和侵袭转移, 在临床肿瘤的诊断、治疗及预后中具有深远意义。研究miRNAs与肿瘤侵袭转移关系, 可以提示我们通过诱导促侵袭转移的miRNAs沉默或诱导抑制侵袭转移的miRNAs高表达而在临床上达到抑制肿瘤侵袭转移的目的。有研究^[51]^表明miRNAs有望成为一些肿瘤早期诊断和判断预后的重要指标。随着人们对miRNAs及其在肿瘤侵袭转移中的研究不断深入, 未来miRNAs必将为临床上肿瘤的诊断、治疗及预后判断提供新的方法。
